# Systemic Neutralizing Antibodies and Local Immune Responses Are Critical for the Control of SARS-CoV-2

**DOI:** 10.3390/v14061262

**Published:** 2022-06-10

**Authors:** Shaswath S. Chandrasekar, Yashdeep Phanse, Mariah Riel, Rachel E. Hildebrand, Mostafa Hanafy, Jorge E. Osorio, Sherein S. Abdelgayed, Adel M. Talaat

**Affiliations:** 1 Department of Pathobiological Sciences, School of Veterinary Medicine, University of Wisconsin, Madison, WI 53706, USA; schandrasek5@wisc.edu (S.S.C.); mariah.riel@wisc.edu (M.R.); rhildebrand@wisc.edu (R.E.H.); hanafy@wisc.edu (M.H.); jorge.osorio@wisc.edu (J.E.O.); 2 Pan Genome Systems, Madison, WI 53719, USA; phanse.yashdeep@gmail.com; 3 Department of Microbiology and Immunology, Faculty of Veterinary Medicine, Cairo University, Giza 12211, Egypt; 4 Colombia Wisconsin One Health Consortium, Universidad Nacional Medellín, Calle 75#79a 5, Colombia; 5 Department of Pathology, Faculty of Veterinary Medicine, Cairo University, Giza 12211, Egypt; sherein.abdelgayed@cu.edu.eg

**Keywords:** SARS-CoV-2, COVID-19, mucosal immunity, correlate of protection, nanovaccine

## Abstract

Antibody measurements are primarily used to evaluate experimental and approved COVID-19 vaccines, which is unilateral considering our immune responses’ complex nature. Previously, we showed that nanoparticle plasmid DNA adjuvant system, QAC, and MVA based vaccines were immunogenic against SARS-CoV-2. Here, we report on the protective efficacy of systemic humoral and mucosal cell-mediated immune responses in transgenic mice models against SARS-CoV-2 following nanoparticle immunization. Parenteral, intramuscular administration of QAC-based plasmid DNA vaccine-encoding SARS-CoV-2 S and N led to the induction of significant serum neutralizing humoral responses, which reduced viral burden in the lungs and prevented viral dissemination to the brain. In contrast, the mucosal, intranasal administration of a heterologous vaccine elicited significant mucosal cell-mediated immune responses in the lungs that limited lung viral replication. The presented results demonstrate that serum neutralizing humoral and local lung T-cell immune responses are critical for the control of SARS-CoV-2 replication.

## 1. Introduction

The COVID-19 pandemic caused by the severe acute respiratory syndrome Coronavirus-2 (SARS-CoV-2) has presented a significant challenge. Several nucleic-acid, viral vector and subunit vaccines have been authorized for use worldwide to help limit the spread of SARS-CoV-2 and the severity that is associated with COVID-19 [1,2,3]. Vaccines were found to be highly effective and, in some instances, >95% effective at reducing hospitalization and death that was associated with COVID-19 caused by the original lineage A virus [1,2]. However, with the emergence of new variants, such as the more transmissible β and δ variants, additional strategies are required to limit transmission. Although using a 2nd booster shot for the mRNA-based vaccines has been shown to boost neutralizing antibody (nAb) responses against the β and δ variants, new variant-specific vaccines might be used for the potentially immune-evading omicron variant [4,5,6]. A lengthy approval process with large clinical trials for variant-specific vaccines using the same approved platform technology can be circumvented by identifying relevant correlates of protection against SARS-CoV-2. In this study, we have shown that systemic neutralizing antibodies and mucosal T-cell responses are relevant correlates of protection for COVID-19 post-vaccination.

For several viral diseases, systemic nAbs are widely accepted as the most relevant correlate of protection, although this is possibly due to the ease of measuring this immunological parameter. For example, for smallpox, a 1:20 nAb titer is protective, but CD8+ T-cells in the skin are essential for preventing poxvirus growth in the skin [7]. Antibodies in the gut of vaccinated individuals against poliomyelitis protected better against reinfection with oral poliovirus infection [8]. Similar relevant correlates of protection need to be identified for SARS-CoV-2 in order to identify surrogate markers of protection to potentially accelerate vaccine approval in the future. Although humoral and CD4+ T-cell immunity is essential to prevent early infection of viral pathogens, CD8+ T-cells might be necessary for controlling replication once the infection has been established and they are essential in reducing the severity of the disease [9]. The role of CD8+ T-cell mediated immunity is gaining even more relevance because approved COVID-19 vaccines reduce the severity that is associated with lineage B.1.351 SARS-CoV-2 infection, despite poor nAb responses against the variant [10]. Furthermore, mucosal antibody responses, such as dimeric mucosal IgA and resident T-cells can limit pathogenic human coronavirus infection in the upper and lower respiratory tract [11]. Similarly, studies investigating relevant corelates of protection should also take into consideration mucosal and systemic T-cell responses. Although effective, mucosal responses are seldom induced by parenteral vaccine administration but require mucosal routes of administration, such as the intranasal route that we have attempted to study, described here.

The ability of T-cells and mucosal immunity to protect against SARS-CoV-2 needs to be thoroughly characterized if we are to develop better vaccines. In this study, we investigated the protective efficacy of mucosal T-cell and systemic nAbs using different vaccination platforms and routes against SARS-CoV-2 infection in transgenic mice. Quil-A chitosan (QAC) encapsulation of plasmid DNA immunogens protects against nuclease activity and enables the delivery of DNA vaccines via the mucosal route [12]. We have previously shown that QAC based nucleocapsid vaccines (pQAC-IBV) can elicit protective CD8+ T-cell responses against avian coronavirus [12]. Poxviruses, such as modified vaccinia ankara (MVA) viral vector-based SARS-CoV-2 vaccines that are shown by us and others are safe, well-tolerated, and protect transgenic mice and rhesus macaques against SARS-CoV-2 infection and pathology [13,14]. In this report, the parenteral, intramuscular (IM) administration of 2-dose QAC-encapsulated plasmid DNA (pQAC-CoV) induced systemic nAbs that protected against viral replication in the lungs and brain. In contrast, the intranasal (IN), mucosal heterologous administration of pQAC prime-followed MVA vector (MVA-CoV) led to the induction of SARS-CoV-2 specific lung type-1 CD8+ and CD4+ T-cell responses. These mucosal responses provided local protection by reducing viral replication in the lungs, but they did not prevent viral dissemination to the brain. Our results highlight that both mucosal lung and systemic immune responses can limit SARS-CoV-2 replication. 

## 2. Materials and Methods

### 2.1. Cells and Viruses

HEK 293T cells and Vero E6 cells were a kind gift from Dr. Jorge Osorio and were maintained in DMEM that was supplemented with 10% fetal bovine serum (FBS) and penicillin-streptomycin (D10) at 37 °C in a 5% CO_2_ atmosphere. J774 cells were maintained in RPMI that was supplemented with 10% fetal bovine serum (FBS) and penicillin-streptomycin (D10) at 37 °C in a 5% CO_2_ atmosphere.

### 2.2. Preparation of SARS-CoV-2 Vaccine Constructs

QAC and MVA-based SARS-CoV-2 vaccine constructs were developed as described previously [13]. Sequences for the SARS-CoV-2 nucleocapsid (N) were downloaded (GenBank accession number MN908947), back-translated, and codon-optimized for expression in mice. The full-length SARS-CoV-2 N gene with 6X-His tag added to the 3′ end was developed for immunization. Vector pCAGGS containing the SARS-related Coronavirus 2 Wuhan-Hu-1 spike glycoprotein gene (soluble, stabilized), NR-52394 was obtained through BEI Resources, NIAID, NIH. The spike construct that was used for immunization was soluble and it was deficient in the furin cleavage site (S1–S2 cleavage deficient) with 2P mutations in the heptad repeat (HR) region 1 to stabilize the glycoprotein. To confirm the insertion of genes in the correct orientation, DNA sequencing was performed at the UW-Madison Biotechnology Center with an ABI Prism 3730XL DNA analyzer using BigDye terminators (Applied Biosystems, San Francisco, CA, USA). The MVA expressing N and S constructs was generated as described before in CEF cells [15]. Plasmid-loaded QAC particles were synthesized as described previously [12].

### 2.3. Characterization of Nanoparticles

The size distribution and zeta potential of QAC-NPs in aqueous dispersion was measured by dynamic light scattering (DLS) on a Malvern Zetasizer instrument at 25 °C. For size distribution, 10 ul of QAC-NPs in solution was diluted to 3 mL using nuclease-free water and placed in a low volume cuvette and analyzed directly. For the zeta potential measurement, approximately 1 mL of the diluted QAC-NPs in solution was placed in a disposable capillary zeta potential cell that was available from the Zetasizer Nano series. For the release kinetics assay, QAC NPs loaded with 50 ug total DNA was resuspended in 50 µL of 0.05 M phosphate-buffered saline (PBS, pH 7.4) at 37 °C in duplicates. At each time point, the suspensions were removed and centrifuged at 14,000 rcf for 20 min. The supernatant was removed and replaced with PBS and returned to incubation. The supernatant samples were quantified for released DNA from the QAC using a GE Healthcare/Amersham Biosciences Ultrospec 3100 Pro UV/Visible Spectrophotometer and compared to the total DNA that was used. For the delivery experiment, pQAC-Luc was added to HEK 293T cells in 96-well plate format at different DNA amounts listed. At 72 h post addition, the cells were lysed, and luciferase activity was measured using the ONE-Glo™ Luciferase Assay System (Promega, Madison, WI, USA). RLU were measured using the TD 20/20 Luminometer (Turner Designs, San Jose, CA, USA). For stability studies, HEK 293T cells seeded in 96-well format were transfected with fresh or released pCAG-Luc in the supernatant (24 h) from the release kinetics assay using TransIT^®^-293 Transfection Reagent, according to the manufacturer’s instructions (Mirus Bio, Madison, WI, USA). Three days post-transfection the cells were lysed and luciferase activity was measured using the ONE-Glo™ Luciferase Assay System (Promega, Madison, WI, USA). RLU were measured using the TD 20/20 Luminometer (Turner Designs, San Jose, CA, USA).

### 2.4. J774 Uptake Experiment

To evaluate the internalization of QAC-NPs, J774 cells (Mus musculus macrophage cells) were plated in at a density of 0.5 × 10^6^ cells/mL in a 24-well plate with coverslips and incubated overnight at 37 °C. The following day, the cells were incubated with Cy3-labeled plasmid DNA (Label IT^®^ Plasmid Delivery Controls, MIR7904) that was encapsulated by QAC NPs (2 ug/well) for 4 h or 24 h. The cells were then washed with phosphate-buffered saline (PBS, pH 7.4) to remove non-adherent or loosely adherent NPs and fixed in 4% paraformaldehyde (methanol free). The cells were permeabilized with 0.1% Triton X-100 in PBS for 3 min and washed with PBS. Actin staining was performed by incubating the cells with Alexa Fluor 647 Phalloidin (Life Technologies, Grand Island, NY, USA) for 20 min in PBS at room temperature. Coverslips containing the stained cells were washed and mounted on glass slides using a ProLong with DAPI (Life Technologies, Grand Island, NY, USA). Confocal microscopy was performed using an inverted Olympus Fluoview 1000 laser scanning microscope. Final images were prepared using Image J v1.47m software (NIH, Bethesda, MD, USA).

### 2.5. Vaccine Efficacy Study

The efficacy of the experimental vaccine constructs was evaluated in K18-hACE2 mice (6 weeks of age) that were obtained from The Jackson Laboratory and maintained in bio-safety level-2 (BSL) containment pre-challenge and BSL-3 post challenge. At every indicated time point the mice were concurrently immunized with MVA or pQAC S and N constructs. In the primary trial, a total of 60 mice was divided equally into four groups (*n* = 15 each). The groups of mice were either unvaccinated (PBS) or immunized with pQAC-CoV (IM) or pQAC-CoV (IN) at week 0, and week 6. Another group of K18-hACE2 mice was vaccinated with pQAC-CoV (IN) at week 0, followed by a boost with MVA-CoV (IN) at week 6. A vaccine dose of 50 μg/ plasmid DNA construct/animal, and 10^8^ pfu/ MVA construct/animal was administered at each immunization time point. Sera for neutralizing antibody titers were harvested from blood that was collected at week 9. At week 9, three weeks post-final boost and pre-challenge, the mice (*n* = 4) were euthanized, BAL was collected as described previously in D10 media, and their lungs were harvested and processed for ICS assay as described below. At week 9, the mice were challenged with SARS-CoV-2 isolate USA-WA1/2020 intranasally at a dosage of 10^4^ PFU. The mice were weighed on the day of the SARS-CoV-2 challenge and everyday thereafter. A total of 5–6 mice were euthanized at 4- and 6-days post-challenge (dpc). The mice were euthanized via isoflurane overdose and then cervical dislocation. Lung, spleen, and brain were collected for viral load quantitation and histopathology. A second follow-up study was conducted to validate the findings of the initial trial. In the follow-up trial, a total of 24 mice was divided equally into two groups (*n* = 12 each). The groups of mice were either unvaccinated (PBS) or immunized with pQAC-CoV (IM) at week 0, and week 6 and challenged as described above at week 9. Samples were collected for vaccine efficacy read outs as detailed above.

### 2.6. SARS-CoV-2 Neutralization Assay

SARS-CoV-2, isolate USA-WA1/2020 (lineage A), or isolate SA/2020 (lineage B.1.351) or isolate Englan/2020 (lineage B.1.1.7), kind gifts from Dr. Jorge Osorio was propagated and titrated on Vero E6 cells. Heat-inactivated sera and BAL were first serially diluted in serum-free Opti-MEM media and incubated with 100 PFU per well of SARS-CoV-2 isolates for 60 min at 37 °C and transferred into wells that were pre-seeded with Vero E6 cells. The plates were incubated at 37 °C for four days before scoring for the cytopathic effect. The neutralization titer was calculated as the reciprocal of the highest dilution at which virus neutralization occurred.

### 2.7. Flow Cytometric Assessment of SARS-CoV-2 Specific Intracellular Cytokine Assay

Immunized K18-hACE2 mice (*n* = 4) from each vaccine group at 3 weeks post-final boost were euthanized and used for flow cytometric assessment. Single-cell suspensions from the lungs that were prepared using standard techniques were used. Briefly, lungs were excised and placed in a gentleMACS dissociator M Tube (Miltenyi 130-093-236) with 3mL collagenase B (1 mg/mL, Roche). Lung tissue was processed using the gentleMACS dissociator, followed by incubation for 30 min at 37 °C. Single-cell suspensions from the lungs were prepared by gently squeezing through a 70-mm cell strainer (Falcon) after lysing RBCs using 1X BD Biosciences BD Pharm Lyse™. For intracellular cytokine staining, 1 × 106 cells were stimulated with SARS-CoV-2 spike protein (BEI re-sources-NR-52396, 100 ng total/well) or N Protein N-terminal RNA binding domain (BEI resources-NR-53246, 100 ng total/well) overnight (~18 h) at 37 °C. Brefeldin A (1 μL/mL, GolgiPlug, BD Biosciences, San Jose, CA, USA) was added after, and the cells were further incubated for another 5 h at 37 °C. Fluorochrome-labeled antibodies against the cell-surface antigens CD4 (BUV 496, GK1.5), CD8a (BUV395, 53-6.7), and intracellular antigens IFN-γ (APC, XMG1.2); TNF-α (BV421, MP6-XT22); IL-2 (PE-CF594, JES6-5H4); IL-17 (FITC, TC11-18H10.1); IL-13 (PE-Cy7, eBio13A); or IL-4 (PerCP-Cy5.5, 11B11) were purchased from BD Biosciences (San Jose, CA, USA); Biolegend (San Diego, CA, USA); eBioscience (San Diego, CA, USA); or Invitrogen (Grand Island, NY, USA). Before antibody staining, the cells were stained for viability with Dye eFluor 780 (eBiosciences, San Diego, CA, USA). After stimulation, the cells were stained for surface markers and then processed with the Cytofix/Cytoperm kit (BD Biosciences, San Jose, CA, USA). To stain for cytokines, the cells were first stained for cell-surface molecules, fixed, permeabilized, and subsequently stained for the cytokines. All samples were acquired on an LSR Fortessa (BD Biosciences, San Jose, CA, USA) flow cytometer. Data were analyzed with FlowJo software (TreeStar, Woodburn, OR, USA). Results are expressed as the difference in the percentage of stimulated cells with that of unstimulated cells. At least 100,000 events were collected for each sample. A Boolean gating strategy was applied for the determination of cytokine-secreting T cells (Figure S1).

### 2.8. Viral Load Measurement

Tissue samples were homogenized to a final 1 mL suspension in serum-free media (Opti-MEM) with sterile zirconia beads, clarified by low-speed centrifugation at 800× *g* for 10 min at 4 °C, and virus titers were determined in Vero E6 cell monolayers that were grown in 96-well plates. The Vero E6 cells were seeded (0.25 × 105/well) in a 96-well plate and incubated overnight at 37 °C in a CO_2_ incubator. A total of 100 µL of 10-fold serially diluted tissue suspension was added to each well in quadruplicate format for 1hr at 37 °C and replaced with fresh complete DMEM media. The plates were incubated in a CO_2_ incubator at 37 °C for 3–4 days, after which the cytopathic effect (CPE) was observed microscopically at 40× magnification. Virus titers were expressed as TCID50 units per gram of tissue and then converted to PFU/mL by multiplying the TCID50/mL by 0.7 [16,17]. For an qRT-PCR, RNA was extracted from homogenized brain samples (see above, 100 µL) using a Zymo Direct-Zol™ RNA mini prep kit (Zymo Research, CA, USA), according to the manufacturer’s instructions. The RT-qPCR was conducted in two steps: cDNA synthesis (Invitrogen™ SuperScript™ III First-Strand Synthesis System) and qPCR reactions. The cDNA synthesis was performed with 0.5 µL (50 ng/µL) random hexamers, 0.5 µL of 10 mM dNTPs, and 4 µL RNA, then heated at 65 °C for 5 min and chilled on ice, followed by the addition of 1 µL of 10X RT buffer, 1 µL of 0.1 M DTT, 1 µL of 25 mM MgCl_2_, 0.5 µL of RNaseOUT and 0.5 µL of SuperScript III enzyme in a final volume of 10 µL. The reaction conditions included 25 °C for 5 min, 50 °C for 60 min and 70 °C for 15 min. A SYBR green RT-qPCR was performed using SARS-CoV-2 *N* gene-specific primer pair set forward primer: 5′ GACCCCAAAATCAGCGAAAT 3′ and reverse primer: 5′ TCTGGTTACTGCCAGTTGAATCTG 3′. PCRs were performed using a StepOnePlus™ Real-Time PCR System (Applied Biosystems, Foster City, CA, USA) under the following conditions: one cycle at 95 °C for 2 min, followed by 40 cycles of 95 °C for 3 s and 60 °C for 30 s. Each 20 µL reaction was carried out using 1 µL of diluted cDNA (1/10), 10 µL of GoTaq^®^ qPCR mastermix (Promega, Madison, WI, USA), 2 µL of forward and reverse primers and 7µL of nuclease free water. A serial 10-fold dilution of cDNA that was extracted from quantitative PCR (qPCR) Control RNA from heat-inactivated SARS-CoV-2, Isolate USA-WA1/2020 (BEI resources, NR-52347) was used to establish the standard curve. A temperature melt curve analysis was used to confirm the specificity of the product.

### 2.9. Histopathological Analysis

Lungs, spleen, and brain that were collected from the different experimental groups were routinely processed. The paraffin embedded blocks were sectioned at 5-micron thickness and stained with hematoxylin and eosin for histopathological examination by a light microscope [18]. For lung histopathological analysis, lung lesions were scored ordinally as follows: 0, absent; 1, minor scattered cells in septa; 2, moderate infiltrates in septa and extending into lumen; or 3, moderate-to-severe infiltrates in septa and lumen with associated consolidation/atelectasis and/or edema [19].

### 2.10. Statistical Analysis

Statistical analyses were performed using GraphPad software (La Jolla, CA, USA). Viral load for the follow-up trial were compared using a Student’s t-test, where *, *p* < 0.05; **, *p* < 0.01; ***, *p* < 0.001; ****, *p* < 0.0001 were considered to be significantly different among groups. Neutralizing antibody titers, cellular immune assays, viral loads and histopathology scoring were compared using a one-way ANOVA test where *, *p* < 0.05; **, *p* < 0.01; ***, *p* < 0.001; ****, *p* < 0.0001 were considered to be significantly different.

## 3. Results

### 3.1. Characterization of Nanoparticles Formed by QAC Encapsulation of Plasmid DNA Immunogens

**Figure 1:**
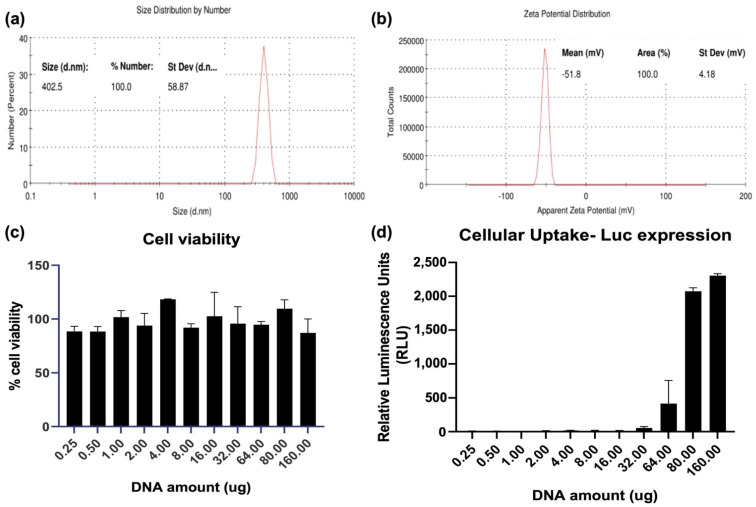

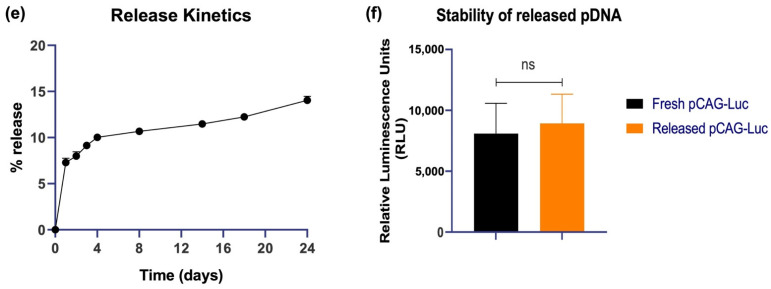
Nanostructure of QAC-encapsulated plasmid DNA. Number-based representative DLS data (**a**) and Zeta potential (**b**) of QAC-SARS-CoV-2 S nanoparticles at 25 °C with Zetasizer software. (**c**) Viability of HEK 293T cells 72 h post addition of increasing amounts of pQAC-Luc as measured using MTT assay. (**d**) Expression of luciferase 72 h post addition of increasing amounts of pQAC-Luc. (**e**) Sustained release kinetics of packaged DNA in vitro measured at pH-7.4, 37 °C. (**f**) Expression of luciferase from released pCAG-Luc used in the release kinetics assay in comparison to fresh pCAG-Luc.

Uptake and the subsequent cellular immune responses that are generated is influenced by the size of the nanoparticle (NP) vaccines [20]. Plasmid DNA encoding for the SARS-CoV-2 spike glycoprotein (pCoV-S) was used for size and charge characterization. QAC complexation of pCoV-S that was suitable for mice inoculation led (pQAC-S) to the formation of nanoparticles around 400 nm using a dynamic light scattering (DLS) analysis post-sonication for disaggregation with a net-positive zeta potential of −51.9 ± 4.58 mV (Figure 1a,b). To evaluate the biocompatibility of QAC-based vaccines in a relevant cell culture model for SARS-CoV-2 vaccines, different amounts of QAC-encapsulated pCAG vector encoding the luciferase gene (pQAC-Luc) was added to HEK 293T cells, and cell viability after 3 days was measured using MTT assay. As expected, no cytotoxicity was observed in the cells post-pQAC-Luc addition even at high DNA amounts, highlighting the safety of QAC-based vaccines (Figure 1c). To quantitatively determine the delivery of cargo plasmid, pQAC-Luc was added to HEK 293T cells in increasing amounts and luciferase expression was assayed 3 days post-addition. The luciferase expression was detected in a dose-dependent manner, indicating the delivery and expression of luciferase from the packaged construct (Figure 1d). The release kinetics of plasmid DNA from pQAC-Luc was evaluated in phosphate-buffered saline (PBS) at physiological temperature (37 °C) and pH (7.4) by quantifying the amount of DNA that was released over time using spectrophotometry. Close to 15% of the packaged pCAG-Luc was released over 24 days in a sustained manner (Figure 1e). To further confirm that the released DNA was functional, pCAG-Luc that was released 24 h after incubation was used to transfect HEK 293T cells with a standard transfection reagent. No difference in luciferase expression was observed between control fresh pCAG-Luc and released pCAG-Luc (Figure 1f). These results underscore the ability of the QAC adjuvant system to release functional and stable plasmid DNA.

### 3.2. Internalization of Plasmid DNA by Macrophages When Complexed with QAC Adjuvant System

**Figure 2:**
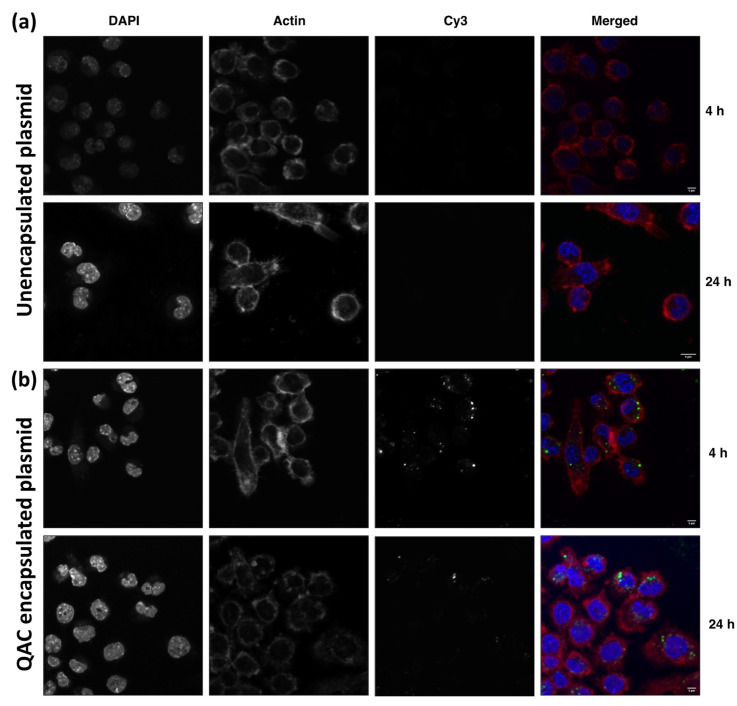
Efficient internalization of QAC nanoparticles by J774 cells. Cell monolayers were incubated with Cy3-labeled (**a**) unencapsulated or (**b**) QAC encapsulated labeled DNA (green) for 4 or 24 h and stained for actin (Alexa phalloidin 546, red). DAPI (blue) was used to stain the nucleus. Representative images were captured by LSCM. Scale bars = 5 μm.

Upon immunization via the multiple routes, vaccine antigens need to be taken up by resident immune cells, such as dendritic cells and macrophages for optimal efficacy [21,22]. The J774 murine macrophages were used to evaluate the ability of QAC to mediate the delivery of plasmid DNA to target immune cells. Murine J774 cells were incubated with fluorescently labeled plasmid DNA that was encapsulated by QAC (pQAC-Cy3) and fluorescent microscopy was used to determine uptake at two different time points (4 and 24 h). These times were chosen to monitor the uptake of the plasmid DNA over time. The Cy3-labeled plasmid DNA was taken up efficiently when delivered by QAC (Figure 2b) which was not observed with the unencapsulated labeled plasmid (Figure 2a). Internalization was observed as early as 4 h post-addition and maintained for 24 h post-addition (Figure 2b). Visually, more labeled plasmid DNA was observed at the 4 h time point than 24 h (Figure 2 and Figure S5). Labeled plasmid DNA was also observed around the nucleus of J774 cells (DAPI stained), indicating favorable localization to the nucleus which should promote the expression of vaccine antigens (Figure 2b). Overall, our analysis indicates that the QAC adjuvant system is well tolerated in cell culture and can mediate the delivery of plasmid DNA to target immune cells.

### 3.3. Systemic SARS-CoV-2 Specific Immune Responses in Vaccinated Mice

**Figure 3:**
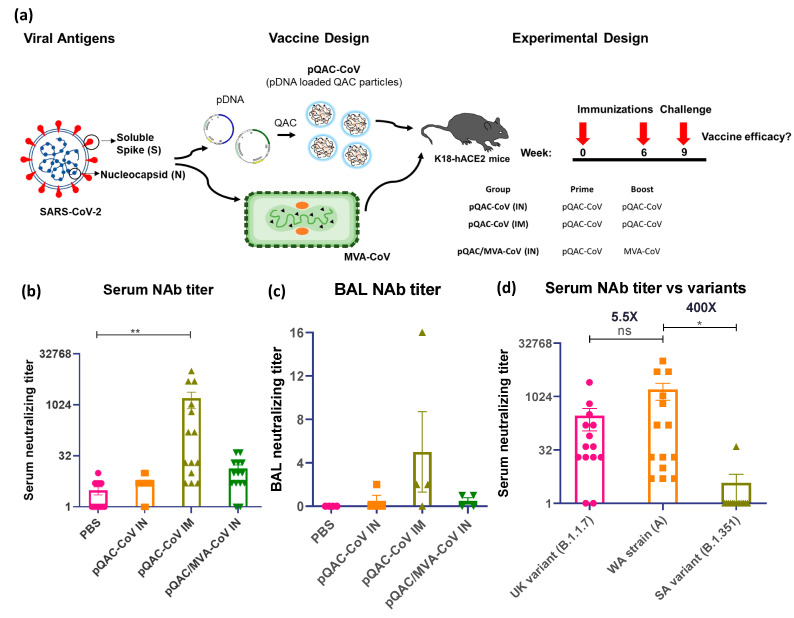
Generation of humoral immune responses in K18-hACE2 mice following immunization with different vaccine constructs. (**a**) Outline for vaccine construct and immunization protocol using groups of K18-hACE2 mice vaccinated with 2 doses of pQAC-CoV (IN) or pQAC-CoV (IM) with 6-week interval. Another group of K18-hACE2 mice were vaccinated with pQAC-CoV (IN) at week-0, followed by boost with MVA-CoV (IN) at week-6. (**b**) Serum neutralization (NAb) titer of wild-type SARS-CoV-2, isolate USA-WA1/2020, (**c**) bronchoalveolar lavage (BAL) neutralization titer of wild-type SARS-CoV-2, isolate USA-WA1/2020 and (**d**) serum NAb titer of wild-type SARS-CoV-2, isolate USA-WA1/2020 in comparison to UK (B.1.1.7) and SA (B.1.351) variants. Significance (*, *p* < 0.05, **, *p* < 0.01) was determined by ANOVA. Data show mean ± SEM.

Groups of K18-hACE2 mice were immunized with QAC-complexed plasmid DNA (pCoV-S and pCoV-N, both termed pQAC-CoV) via IN or IM routes, followed by boosting at 6 weeks post-initial immunization (Figure 3a). The pQAC-CoV parenteral (IM) administration led to the significant induction of SARS-CoV-2 neutralizing antibody titers (nAb) in harvested sera at 3 weeks post-final vaccination (wpv, Figure 3b). The neutralizing antibody levels were also higher with heterologous vaccination where mice were primed by pQAC-CoV IN, followed by MVA-CoV IN administration (pQAC/MVA-CoV) as reported previously, albeit non-significant (Figure 3b). Interestingly, bronchoalveolar lavage (BAL) that was harvested from pQAC-CoV IM-vaccinated mice was able to neutralize SARS-CoV-2 at levels higher than detected with the unvaccinated group (Figure 3c). The ability of sera from pQAC-CoV IM-vaccinated mice to neutralize common circulating variants of SARS-CoV-2, B.1.1.7 and B.1.351 was investigated. Neutralization titers against B.1.1.7 showed no significant difference when compared to the SARS-CoV-2 lineage A virus against which the vaccines are developed (Figure 3d). In contrast, neutralization titers against B.1.351 were non-detectable and significantly lower, an observation also seen with other approved COVID-19 vaccines (Figure 3d). Our results suggest that parenteral (IM) QAC based-immunization is better at inducing significant systemic serum-neutralizing immune responses than local, IN immunization.

### 3.4. Intranasal Administration of SARS-CoV-2 Vaccine Induces Lung Cellular Responses

**Figure 4:**
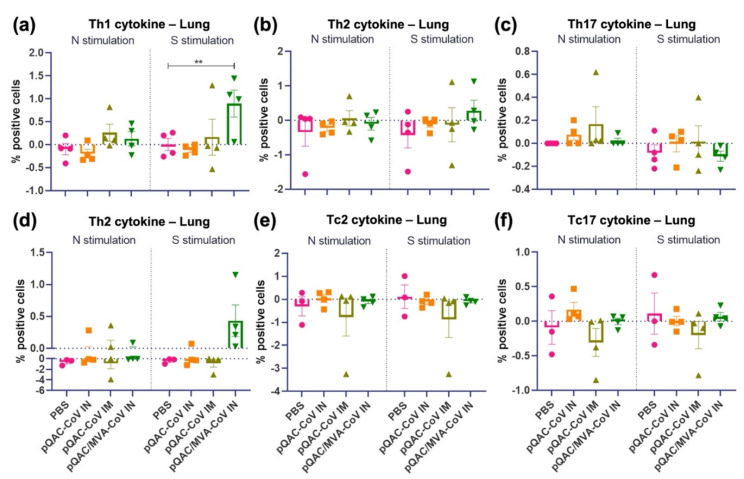
SARS-CoV-2 spike-specific T-cell responses in lungs of vaccinated K18-hACE2 mice. Intracellular cytokine staining was performed on lungs harvested 3 weeks after final boost to assess T-cell responses. (**a**) Type 1 helper (Th1) responses (IFN-γ or TNFα or IL-2+); (**b**) type 2 helper (Th2) responses (IL-13+); (**c**) type 17 helper (Th17) responses (IL-17+); (**d**) type 1 cytotoxic (Tc1) responses (IFN-γ or TNFα or IL-2+); (**e**) type 2 cytotoxic (Tc2) responses (IL-13+); (**f**) type 17 cytotoxic (Tc17) responses (IL-17+) intracellular cytokine staining assays for lung T-cells in response to recombinant SARS-CoV-2 spike stimulation. Significance (**, *p* < 0.01) was determined by ANOVA compared to PBS controls. Data show mean ± SEM.

Intracellular cytokine staining (ICS) was performed with lung cells that were harvested from vaccinated mice three weeks post-final boost (pre-challenge) to evaluate local lung immune responses that were elicited by QAC-based vaccines. For S and N-specific immune responses the cells were stimulated with purified recombinant spike glycoprotein and N Protein N-terminal RNA binding domain from SARS-CoV-2, respectively, overnight before staining. As seen previously, pQAC/MVA-CoV vaccination led to the induction of type 1 helper (Th1, Figure 4a) and cytotoxic (Tc1, Figure 4d) T-cell responses (IFN-γ or TNFα or IL-2+) post-SARS-CoV-2 S stimulation. Expression levels of individual cytokines and representative flow plots can be found in the supplemental information (Figures S2–S4). Detectable immune responses were not elicited by the 2-dose IN administration of pQAC-CoV, potentially highlighting the beneficial impact of mixing and matching vaccination platforms, as seen with pQAC-MVA-CoV (Figure 4a–f). As expected, no induction of deleterious type -2 (IL-4+ or IL-13+) immune responses was observed in the lungs from any of the vaccinated groups (Figure 4b,e). Interestingly, detectable cytokine producing T-cells were only observed with S stimulation and not with N stimulation in any of the vaccinated mice (Figure 4a–f). Further, pQAC-CoV IM administration did not lead to the induction of T-cell immune responses in the lungs (Figure 4a–f). These results indicate that pQAC/MVA-CoV IN administration elicits better local lung immune T-cell responses, in contrast to pQAC-CoV IM administration which elicits better systemic antibody responses.

### 3.5. QAC Based Immunizations Reduce Viral Burden in Transgenic Mice

**Figure 5:**
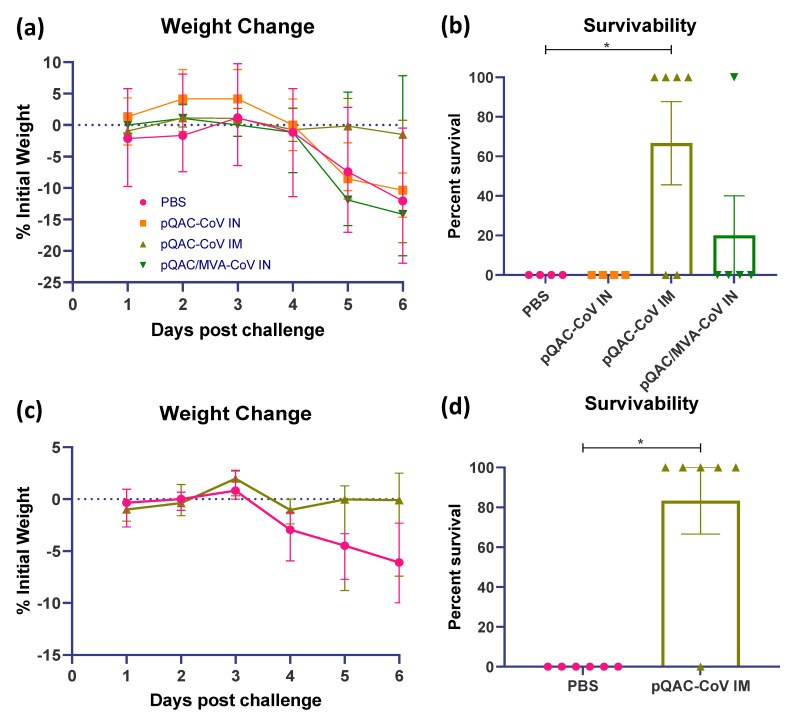
Protective efficacy of QAC-based SARS-CoV-2 vaccines in K18-hACE2 mice. Three weeks following final immunization, K18-hACE2 mice were intranasally infected with 1 × 10^4^ PFU of SARS-CoV-2. (**a**,**c**) Weight loss and (**b**,**d**) survival outcomes at 6 days post-challenge (dpc) in K18-hACE2 transgenic mice. Data from follow-up trial depicted in c and d. Weight loss data show median with error (95% CI). Survivability significance (*, *p* < 0.05) was determined by Mantel–Cox log rank test. Survivability data show mean ± SEM.

**Figure 6:**
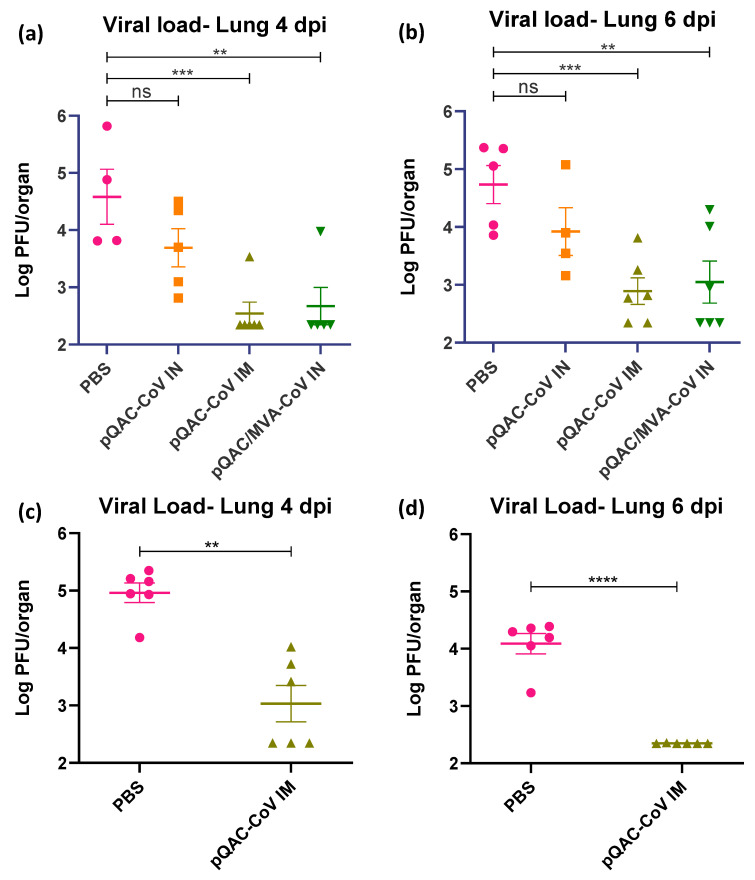
pQAC-CoV administration reduces lung tissue titer. SARS-CoV-2 titers in the lungs of vaccinated mice at 4 (**a**,**c**) and 6 (**b**,**d**) days post-infection (dpi). Data from follow-up trial depicted in (**c**,**d**). Significance (**, *p* < 0.01; ***, *p* < 0.001; ****, *p* < 0.0001) or non-significance (ns) was determined by ANOVA compared to PBS controls (**a**,**b**) or Student’s *t*-test (**c**,**d**). Data show mean ± SEM.

**Figure 7:**
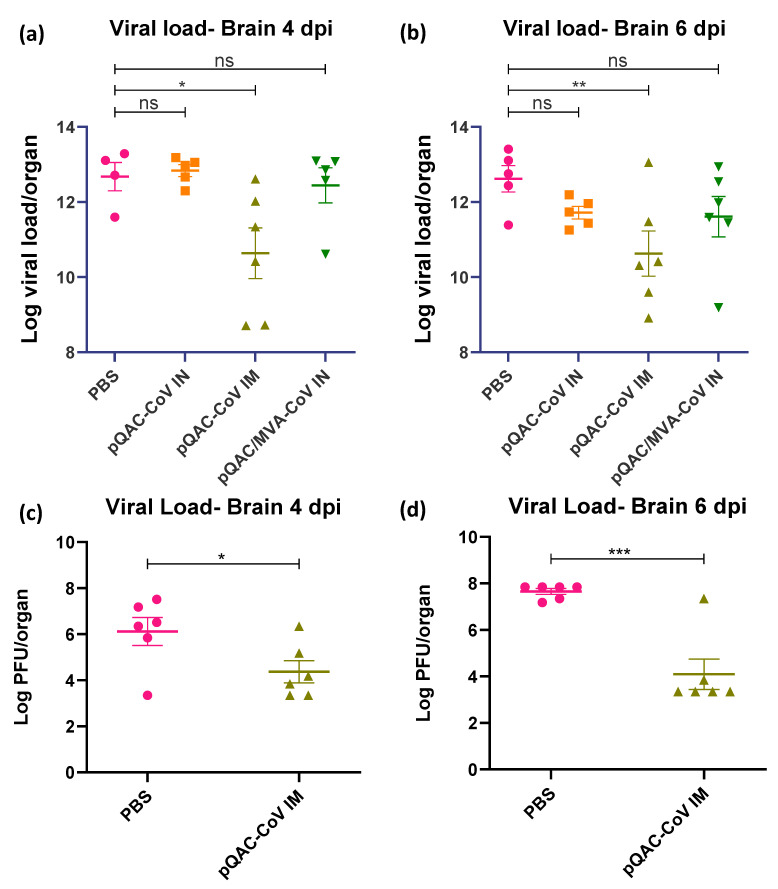
Parenteral pQAC-CoV administration prevents viral dissemination to the brain. SARS-CoV-2 titers in the brains of vaccinated mice at 4 (**a**,**c**) and 6 (**b**,**d**) days post-infection (dpi). Viral titers measured using SARS-CoV-2 specific qRT-PCR (**a**,**b**) or infectious assay using VERO E6 cells (**c**,**d**). Data from follow-up trial depicted in c and d. Significance (*, *p* < 0.05; **, *p* < 0.01; ***, *p* < 0.001) or non-significance (ns) was determined by ANOVA compared to PBS controls (**a**,**b**) or Student’s *t*-test (**c**,**d**). Data show mean ± SEM.

The ability of the experimental QAC-based vaccines to protect K18-hACE2 transgenic mice against SARS-CoV-2 challenge was investigated. Three weeks post-final vaccination, all vaccinated and control (PBS) mice were challenged with a lethal dose of SARS-CoV-2, isolate USA-WA1/2020 (10^4^ pfu/animal) intranasally, against which the vaccines were developed. A follow-up study was conducted with only the PBS and pQAC-CoV IM vaccinations for reproducibility and to validate the findings of the first trial. The outputs from the follow-up trial are depicted in (c,d) of Figure 5, Figure 6 and Figure 7. The unvaccinated mice started losing weight at 4 days post-challenge (dpc) with all mice meeting the criteria for euthanasia by 6 dpc (Figure 5a,c). In contrast, a majority of the pQAC-CoV IM-administered mice (65–80%) survived and showed no apparent clinical signs of SARS-CoV-2 infection with no weight loss (Figure 5b,d). pQAC/MVA-CoV that was administered via the IN route offered minimal protection with significant weight loss observed, comparable to the PBS group, and only 20% of the mice survived at the experimental end point (Figure 5b,d). The mice that were vaccinated with pQAC-CoV IN offered no protection with all the mice following a similar clinical trajectory to the unvaccinated mice (Figure 5a,b). We quantified the infectious viral load in the lungs and brains of challenged mice at two different time points, 4 and 6 dpc. A significant reduction in the lung viral load of both pQAC-CoV IM and pQAC/MVA-CoV IN-vaccinated mice when compared to the unvaccinated mice was observed across both time points (Figure 6a–d). In contrast, only the pQAC-CoV IM vaccinated mice had significantly lower viral RNA and infectious viral loads in the brain (Figure 7a–d). In agreement with the immunology findings, we did not see any significant reduction in the viral loads of pQAC-CoV IN-administered mice both in the lungs and brain (Figure 6 and Figure 7). The parenteral administration of pQAC-CoV which induces significant systemic immune responses provided better protection in transgenic mice when compared to both the mucosal vaccine administrations.

### 3.6. Reduced Viral Pneumonia and Tissue Damage in Vaccinated Transgenic Mice

**Figure 8:**
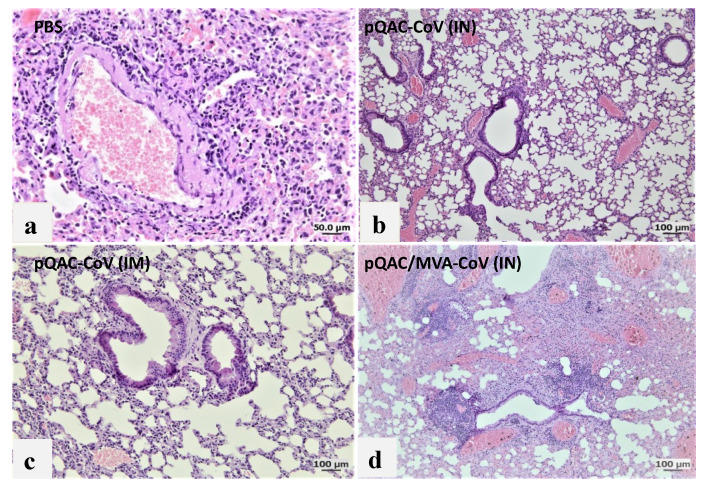

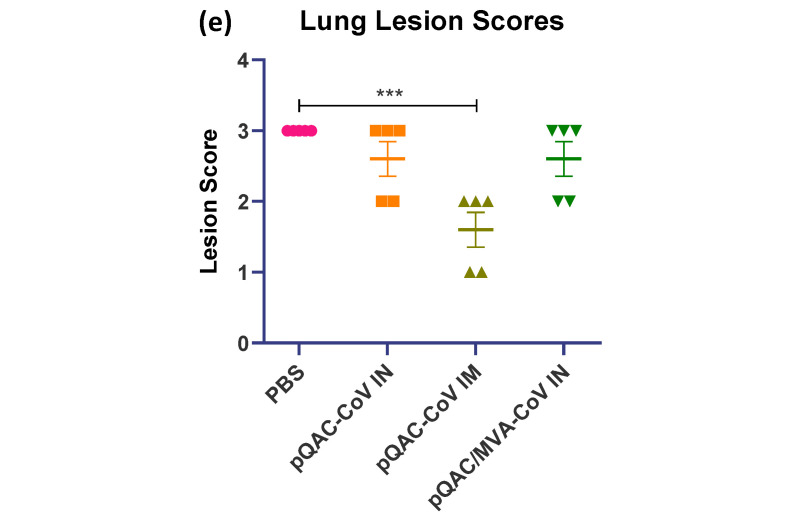
Histopathologic analysis of SARS-CoV-2 infection in K18-hACE2 transgenic mice immunized with QAC-based vaccines. Histology of fixed lung tissues, 6 days after SARS-CoV-2 infection. H&E-stained tissues (*n* = 5 per group). Representative images of SARS-CoV-2-infected mice that received (**a**) PBS, (**b**) pQAC-CoV (IN), (**c**) pQAC-CoV (IM) or (**d**) pQAC/MVA-CoV (IN). Interstitial lung disease was reduced in the pQAC-CoV (IM). Scale bar, 50 or 100 μm. (**e**) Histopathologic scoring of lung tissues. Tissues from all four groups were ordinally scored for lung lesions. Error bars represent the SEM. ***, *p* < 0.001, one-way ANOVA.

To further examine the efficacy of the different experimental vaccines, hematoxylin and eosin (H&E) staining of lung, spleen and brain sections from vaccinated mice at 6 dpc was conducted and histopathological changes were examined in a blinded fashion. Examinations of lungs from unvaccinated mice showed 75% of lung involvement with robust aggregates forming circumferential perivascular lymphoid cuffs and compression of adjacent parenchyma. Furthermore, severe interstitial pneumonia appeared marked as evidenced by mononuclear cells infiltrated the interstitial tissue septa and lumen with consolidation and atelectasis (Figure 8a). Examination of lungs from pQAC-CoV IN-vaccinated mice revealed 25% of lung involvement with a moderate degree of interstitial pneumonia (Figure 8b). In contrast, the pQAC-CoV IM-vaccinated group reported very mild lungs lesions in comparison to the unvaccinated mice with only 10% of lung involvement in the form of very few perivascular solitary lymphoid cell aggregates and mild interstitial pneumonia (Figure 8c). Examination of lungs from the pQAC/MVA-CoV IN-vaccinated group denoted 50% of lung involvement with perivascular lymphoid cuffing, together with less severe interstitial pneumonia than the unvaccinated group (Figure 8d). To quantify the difference in lung severity, the interstitial inflammatory response in each lung sample was scored, including lymphocytic infiltration, extension into the airspaces, and associated edema and atelectasis [19]. As shown in Figure 8e, the pQAC-CoV IM-vaccinated mice showed the least sign of interstitial inflammatory response at 6 dpc. Similar pathology results were noted in the organs of vaccinated mice with few splenic and brain lesions and minimum tissue damage in comparison to the unvaccinated mice (data not shown), which correlates well with the clinical outcome and viral load data. Overall, our analysis indicates that tissue damage was significantly less in mice that were administered the pQAC-CoV via the IM route in comparison to IN vaccination.

## 4. Discussion

Globally, many vaccines have been approved for use against SARS-CoV-2 and are moderately to highly effective in reducing the severity and mortality associated with SARS-CoV-2 infection in humans [1,2,3]. However, with the emergence of new variants, such as B.1.351 and omicron being more common, the ability of the approved vaccines to prevent transmission and limit replication in the upper respiratory tract has reduced [23,24]. Studies investigating variants of concern have shown that at least 93% of T-cell epitopes are conserved across all variants [25]. Therefore, in the absence of nAbs to a specific variant, vaccine-induced T-cell mediated immunity, in theory, could limit replication and subsequent transmission of the variant. Although the approved vaccines effectively generate nAb responses, they are administered parenterally, limiting the induction of local SARS-CoV-2 specific airway epithelium T-cell responses and can be critical in the control of the variant transmission [26]. A positive association has been noted with the presence of SARS-CoV-2 specific T-cells in the BAL of patients with low-to-moderate COVID-19, underscoring the need for mucosal vaccines that can elicit such immunity [27]. In this study, we investigated the protective efficacy of intranasally and parenterally delivered vaccines using the ability of nanoparticle adjuvant system, QAC, and viral vector MVA. The intranasal administration of pQAC/MVA-CoV led to a significant induction of lung type-1 T-cells and low nAb titers that reduced viral burden in the lung post-challenge. In contrast, parenterally administered pQAC-CoV induced systemic nAb responses and limited viral load burden in the lungs and brain, highlighting the ability of QAC-based vaccines to control SARS-CoV-2 replication in transgenic mice.

QAC-based vaccines have been shown previously to be immunogenic and protective against animal and human coronaviruses [12,13]. One of the hallmarks of the QAC adjuvant system is the ability to form nanoparticles (NPs) when complexed with DNA which aids with efficient uptake by immune cells and improves bioavailability [12]. As reported previously, pQAC-CoV that was suitable for mice inoculations formed favorable NPs of around 400 nm. The safety of QAC-based vaccines has been evaluated previously in poultry and mice with no sign of apparent distress noted after immunization [12,13]. Similarly, we did not observe any cytotoxicity of pQAC-Luc vaccine encapsulating pCAG-Luc in cell culture even at high amounts of pQAC-Luc that was added. The above is an interesting observation since other experimental adjuvants incorporating surfactants like quil-A, which is part of QAC, are cytotoxic to some degree in cell culture, thus reinforcing the safety of the QAC adjuvant system [28]. Delivery of packaged cargo was observed in a dose-dependent manner, with an increased expression of luciferase seen with increasing amounts of pQAC-Luc in cells. Similar to our previous study, the QAC adjuvant system was shown to mediate the sustained release of packaged DNA, maintained for up to 24 days [12]. Moreover, the released DNA was also shown to be stable with no effect on its functionality. Antigen uptake by macrophages and dendritic cells (DCs) at the inoculation site is the first of several downstream cellular events that are required for an effective immune response [20]. Mechanisms, such as phagocytosis and pinocytosis are used by target immune cells where the upper particle size limit for internalization is about 0.5 micron [29]. Furthermore, cationic particles, such as QAC NPs, whose charge is primarily imparted by chitosan, are efficiently taken up by macrophages and DCs [29]. As expected, QAC delivered the encapsulated labeled plasmid DNA directly to murine macrophages at different time points. The labeled plasmid DNA was also localized to the nucleus as early as 4 h after addition, which would be required to express vaccine antigens in vivo. Visually, more plasmid DNA was observed at the earlier point (4 h) vs. the later point (24 h), indicating that the internalization and nuclear localization of packaged cargo are rapid. The reduction in fluorescence at a later time point could indicate degradation of the dye as a consequence of plasmid DNA processing or a reduction in florescence intensity after plasmid DNA diffusion from the NPs. These studies highlight the potential mechanism of QAC action in vivo, either directly delivering packaged DNA to target immune cells and/or acting as an antigen depot extracellularly and releasing DNA over time.

Previously, we have shown that a DNA/virus heterologous vaccine approach utilizing pQAC priming, followed by MVA boosting, elicited mucosal T-cell responses and systemic SARS-CoV-2 nAb responses. Interestingly, we did not observe significant systemic antibody responses when we tested our parenteral IM pQAC-CoV vaccine previously. In this study, we tweaked two critical parameters to improve pQAC-CoV’s efficacy. First, we used a spike immunogen that was locked in its prefusion state with a deletion of the poly basic cleavage site with further stabilizing mutations. Previously, we used an unstable spike immunogen without any modifications, which could have compromised vaccine efficacy. Stabilization of the prefusion spike increases the expression of recombinant protein and exposes biologically relevant neutralizing epitopes, improving immune responses [30,31]. Next, we increased the time interval between vaccine doses (six weeks here vs. three weeks, previously). Unpublished studies with QAC vaccines show that delaying booster shots improves vaccine performance, presumably due to the ability of QAC vaccines to sustain and complete DNA release over a prolonged period. With the changes, we noted that parenteral IM administration of pQAC-CoV elicited significantly higher neutralizing antibodies (nAb) in sera than in the unvaccinated mice. We focused on understanding the biologically relevant humoral nAb responses, important for limiting SARS-CoV-2 binding and entry, in contrast to anti-N humoral responses which do not correlate with neutralizing immune responses [32]. As reported previously, pQAC/MVA-CoV also elicited modest serum nAb titers, albeit non-significant [13]. Systemic neutralizing IgG has shown to accumulate in mucosal sites by transuding or passive transfer, which can explain the modest neutralization of lineage A SARS-CoV-2 that was seen with the BAL that was harvested from pQAC-CoV IM-vaccinated mice [33]. Serum nAbs that were elicited by parenteral IM administration of pQAC-CoV effectively neutralized B.1.1.7 variant with a non-significant reduction, compared to the neutralization of the lineage A virus. In contrast, none of the sera was able to neutralize the B.1.351 variant. The B.1.1.7 variant has multiple mutations in the spike, although most of the mutations (N501Y, P681H, D614G) only increase affinity towards the hACE2 receptor [34]. Vaccine-induced nAb responses, as seen here and reported by others are targeted against the S receptor-binding domain (RBD) and should also neutralize the UK strain (only one mutation in RBD, N501Y) efficiently [35]. The B.1.351 variant has multiple mutations in the S RBD (K417N, E484K, N501Y) which can help it to evade monoclonal antibody (mAb) or convalescent sera treatment [36]. It is likely that nAbs that are elicited by QAC-based spike vaccines are directed against the S RBD, thereby neutralizing and conferring protection against lineage A SARS-CoV-2. 

Impaired CD8+ T-cell responses have been linked to increased severity post-SARS-CoV-2 infection and COVID-19 onset, especially in older patients [37]. Although many studies have investigated vaccine-induced T-cell responses, they evaluate only a limited T cell profile, primarily IFN-γ secreting type-1 responses. Here, we looked at a broader profile of lung T-cells, cytotoxic (CD8+, Tc) and helper (CD4+, Th); type-1 (IFN-γ, or TNFα, or IL-2); type-2 (IL-4 or IL-13); and type 17 (IL-17) responses. Heterologous vaccine administration, as reported previously by our group, led to the induction of significant SARS-CoV-2 S-specific Tc1 and Th1 immune responses in the lungs of vaccinated mice [13]. In contrast, no N-specific immune responses were noted in any of the vaccinated groups, potentially due to using just the N Protein N-terminal RNA binding domain for stimulation and not the complete protein like we used for assaying the S-specific responses. Type-1 T-cell responses protect against viral infections in contrast to type-2 immune responses, which can lead to vaccine-associated enhanced respiratory disease (VAERD) [38,39,40,41,42,43,44,45]. No type-2 cytokines secreting T-cells were detected in the lungs of any of the vaccinated mice. Interestingly, the heterologous vaccination was more immunogenic than homologous pQAC-CoV IN, highlighting the advantage of mixing vaccine platforms. Our prior studies indicate that MVA-CoV vaccination alone is not sufficient for generating mucosal T-cell and humoral responses, necessitating the need for pQAC-CoV priming for eliciting robust immune responses [13]. The pQAC-CoV IN immunization in this study did not elicit significant lung T-cell responses, compared to observations with our previous study where the same constructs elicited lung Tc1 responses. The pQAC-CoV IN strategy that was used in the previous study was administered as three doses, compared to the prime-boost regimen that was used in this study, potentially explaining the difference in observed immunogenicity. Overall, our analysis of immune responses in this study indicates that pQAC-CoV IM leads to significant nAb humoral responses. In comparison, IN pQAC/MVA-CoV immunization induced lung T-cell responses with modest systemic nAb responses.

Post SARS-CoV-2 lethal challenge, no weight reduction and ~80% survivability was observed in mice that were administered the parenteral pQAC-CoV vaccine. In contrast, only ~20% survivability was observed with mucosal heterologous vaccination, despite the presence of SARS-CoV-2 specific T-cells in the lungs of vaccinated mice. Interestingly, although minimal protection in terms of clinical outcome was observed with heterologous vaccination, a significant reduction in viral burden in the lungs at different time points (4 and 6 dpc) was noted in comparison to the unvaccinated mice. This highlights the ability of either the lung resident type-1 T-cells or modest nAb titers that are elicited by pQAC/MVA-CoV to limit local lung viral replication. In contrast, a reduction in viral load in the brain was observed only with pQAC-CoV IM administration, necessitating the need for systemic nAbs to prevent viral progression to the brain in K18-hACE2 mice. As reported previously, virus specific T-cells cannot prevent viral entry and initial infection but can limit replication at the lower respiratory tract [9]. Although viral lung burden was reduced in pQAC/MVA-CoV-vaccinated mice, clinical outcomes were not favorable, which might be attributed to the shortcoming of the K18-hACE2 mouse models for evaluating SARS-CoV-2 pathogenesis. The clinical outcomes after SARS-CoV-2 infection in K18-hACE2 mice are linked to neurological rather than respiratory disease, as seen in humans [46]. SARS-CoV-2 replication in the brain is thought to progress via the olfactory system and is independent of viral replication in the lungs of mice [46]. Evaluation of pQAC/MVA-CoV mucosal vaccination in more relevant respiratory disease models of SARS-CoV-2, such as hamsters, ferrets, or non-human primates might yield more conclusive results and better clinical outcomes post-pQAC/MVA-CoV vaccination [47]. However, pQAC-CoV IM administration and the corresponding systemic nAbs also reduced brain viral burden in mice by potentially limiting viral replication in the upper (nasal turbinate or olfactory system) and lower (lung) respiratory tract and reducing viral dissemination to the brain. However, this is yet to be tested directly and the role of lung T-cells and systemic nAbs in mediating protection against SARS-CoV-2 in isolation needs to be further validated. Recent studies have shown the ability of vaccine-induced mucosal T-cells in mediating protection against SARS-CoV-2 variants even in the absence of complementing nAbs, similar to our studies, underscoring their importance for SARS-CoV-2 control [48]. In line with our clinical and viral load data, reduced viral pneumonia in the lungs of pQAC-CoV IM-vaccinated mice were observed. Overall, our results indicate that lung T-cells and systemic neutralizing antibodies can potentially reduce viral replication in the lungs, as seen with pQAC/MVA-CoV and pQAC-CoV vaccinations. Both systemic neutralizing antibodies and mucosal T-cell responses are potentially implicated in controlling respiratory replication and might be relevant correlates of protection against SARS-CoV-2.

## Data Availability

All data are available on request. Please contact: adel.talaat@wisc.edu.

## Supplementary Materials

The following are available online at https://www.mdpi.com/article/10.3390/v14061262/s1, Figure S1: Representative gating strategy for flow cytometry data; Figure S2: SARS-CoV-2 specific CD4+ T-cell responses in lungs of vaccinated K18-hACE2 mice; Figure S3: SARS-CoV-2 specific CD8+ T-cell responses in lungs of vaccinated K18-hACE2 mice; Figure S4: Representative flow plots; Figure S5: Efficient internalization of QAC nanoparticles by J774 cells, 20X magnification.
