# Supplementary material for: Systemic Neutralizing Antibodies and Local Immune Responses Are Critical for the Control of SARS-CoV-2
Source: Viruses. 2022 Jun 10;14(6):1262. doi: 10.3390/v14061262 (PMC9227431; doi:10.3390/v14061262)
Supplement: Supplementary file 1 [file viruses-14-01262-s001.zip › viruses-1739727-SI.pdf]

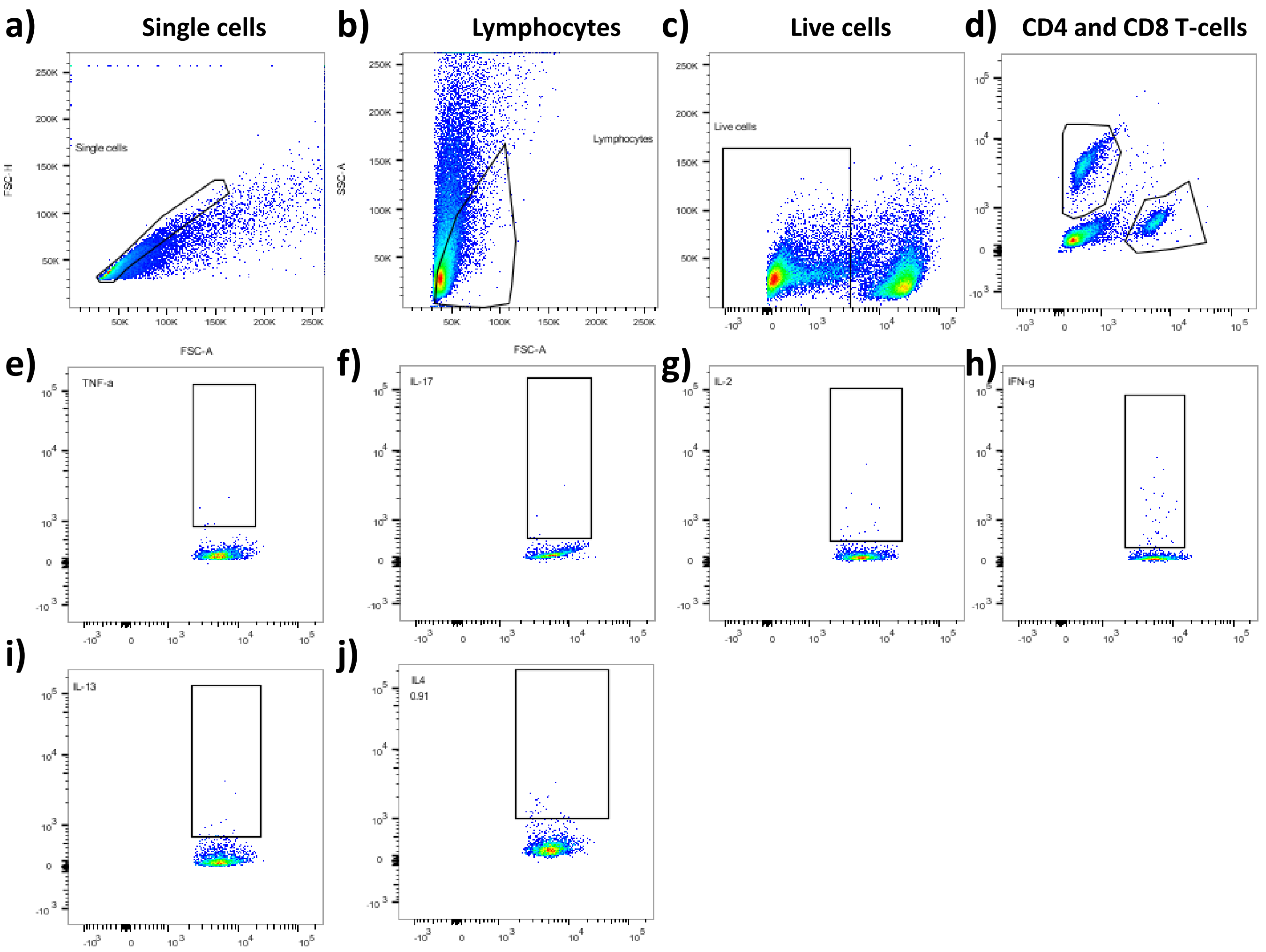

Supplementary Figure S1. Representative gating strategy for flow cytometry data All had the following common gating: a) single cells, b) lymphocyte gate, c) live cells, d) CD4+ and CD8+ T-cells, e) CD4+ TNFα+ cells, f) CD4+ IL-17+ cells, g) CD4+ IL-2+ cells, h) CD4+ IFNγ+ cells, i) CD4+ IL-13+ cells and j) CD4+ IL-4+ cells.

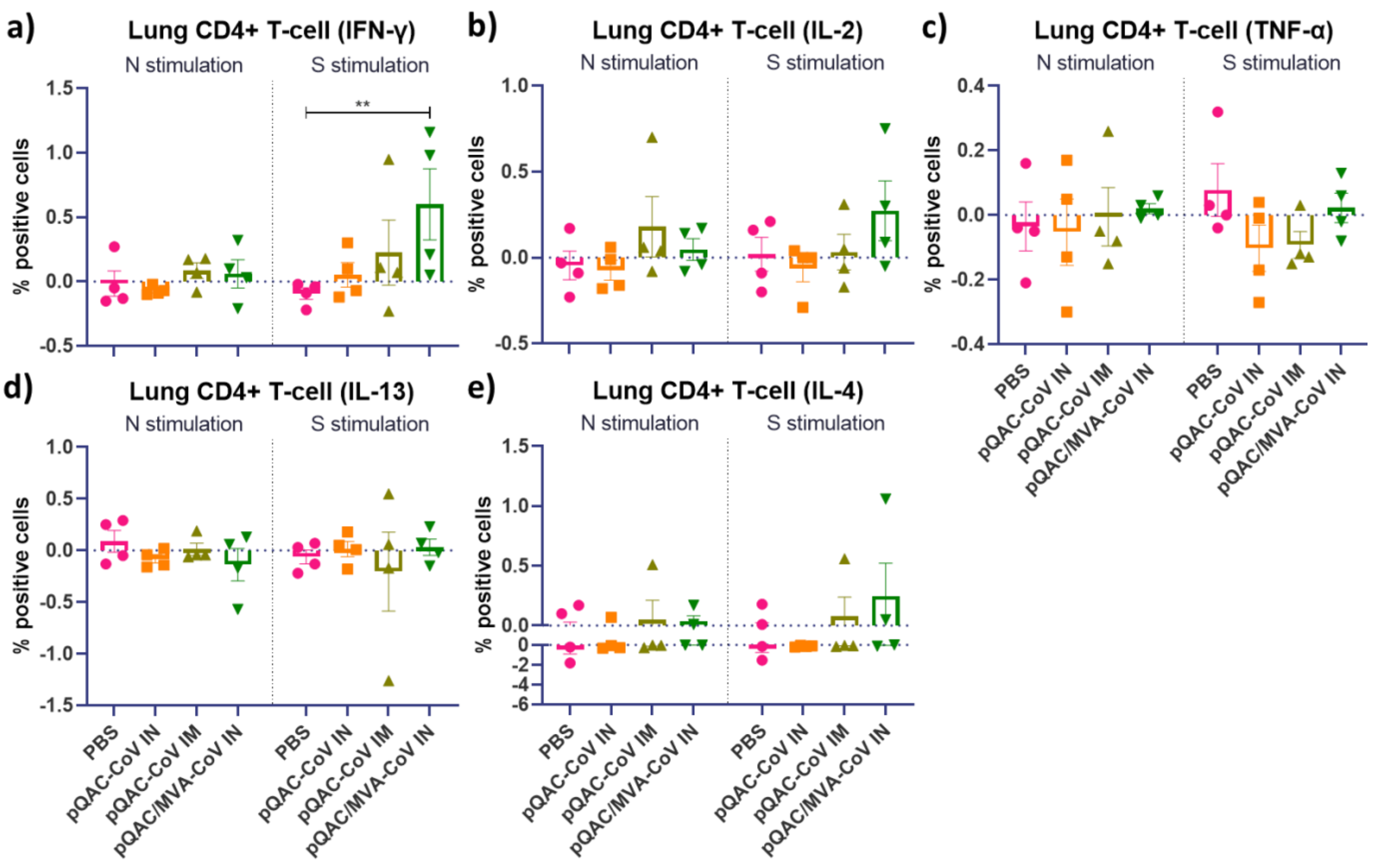

Supplementary Figure S2. SARS-CoV-2 specific CD4+ T-cell responses in lungs of vaccinated K18-hACE2 mice. Intracellular cytokine staining was performed on lungs harvested 3 weeks after final boost to assess CD4+ T-cell responses. (a) IFN-γ, (b) IL-2, (c) TNFα+, (d) IL-13+, (e) IL-4+ T-cells in response to recombinant SARS-CoV-2 protein stimulation.

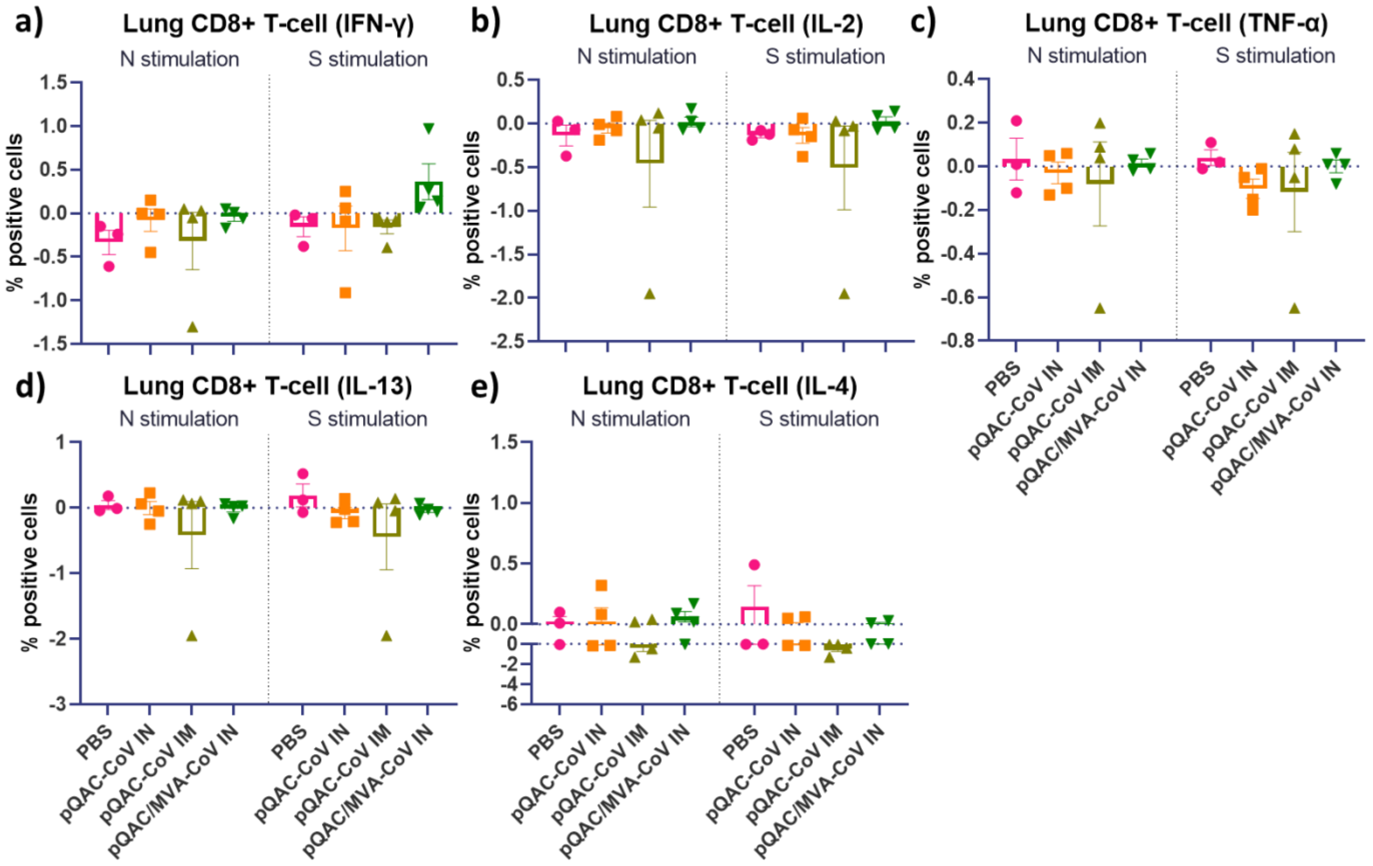

Supplementary Figure S3. SARS-CoV-2 specific CD8+ T-cell responses in lungs of vaccinated K18-hACE2 mice. Intracellular cytokine staining was performed on lungs harvested 3 weeks after final boost to assess CD8+ T-cell responses. (a) IFN-γ, (b) IL-2, (c) TNFα+ , (d) IL-13+, (e) IL-4+ T-cells in response to recombinant SARS-CoV-2 protein stimulation.

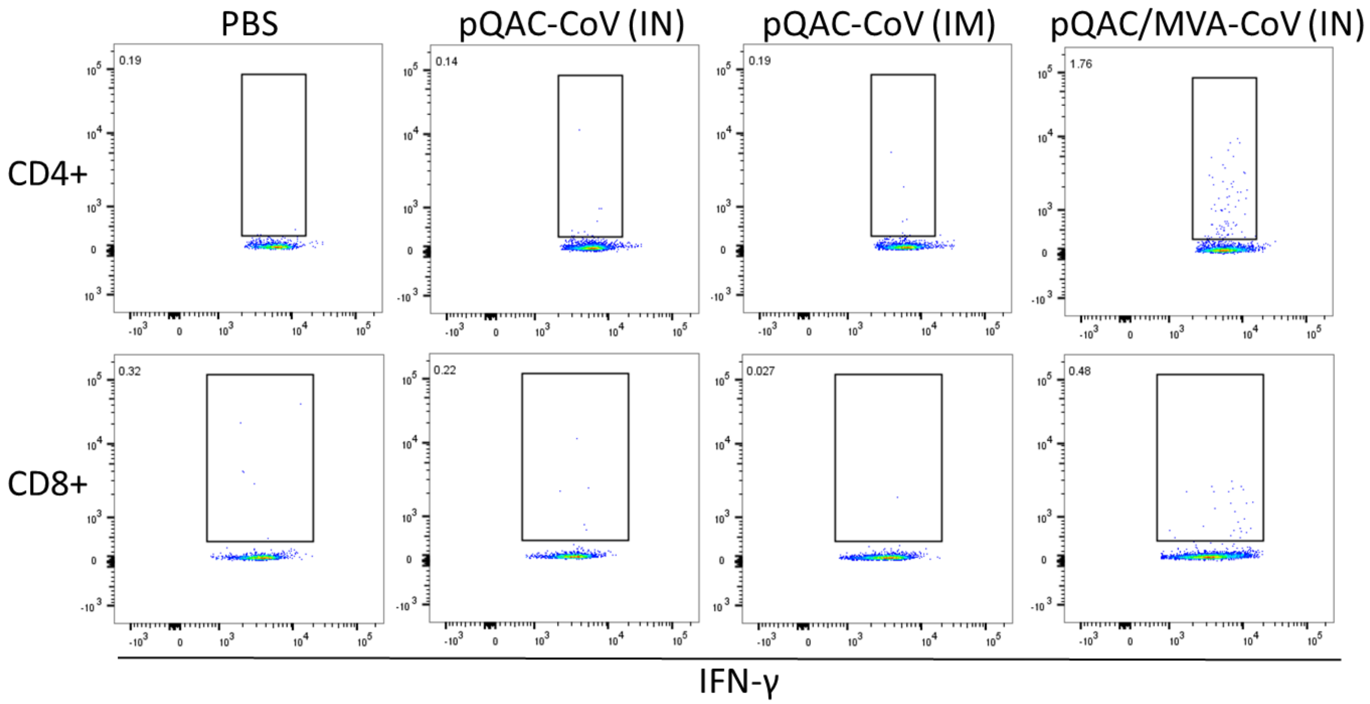

Supplementary Figure S4. Representative flow plots. FACS plots show percentages of IFN- γ secreting CD4+ (top) and CD8+ (bottom) T cells from vaccinated mice.

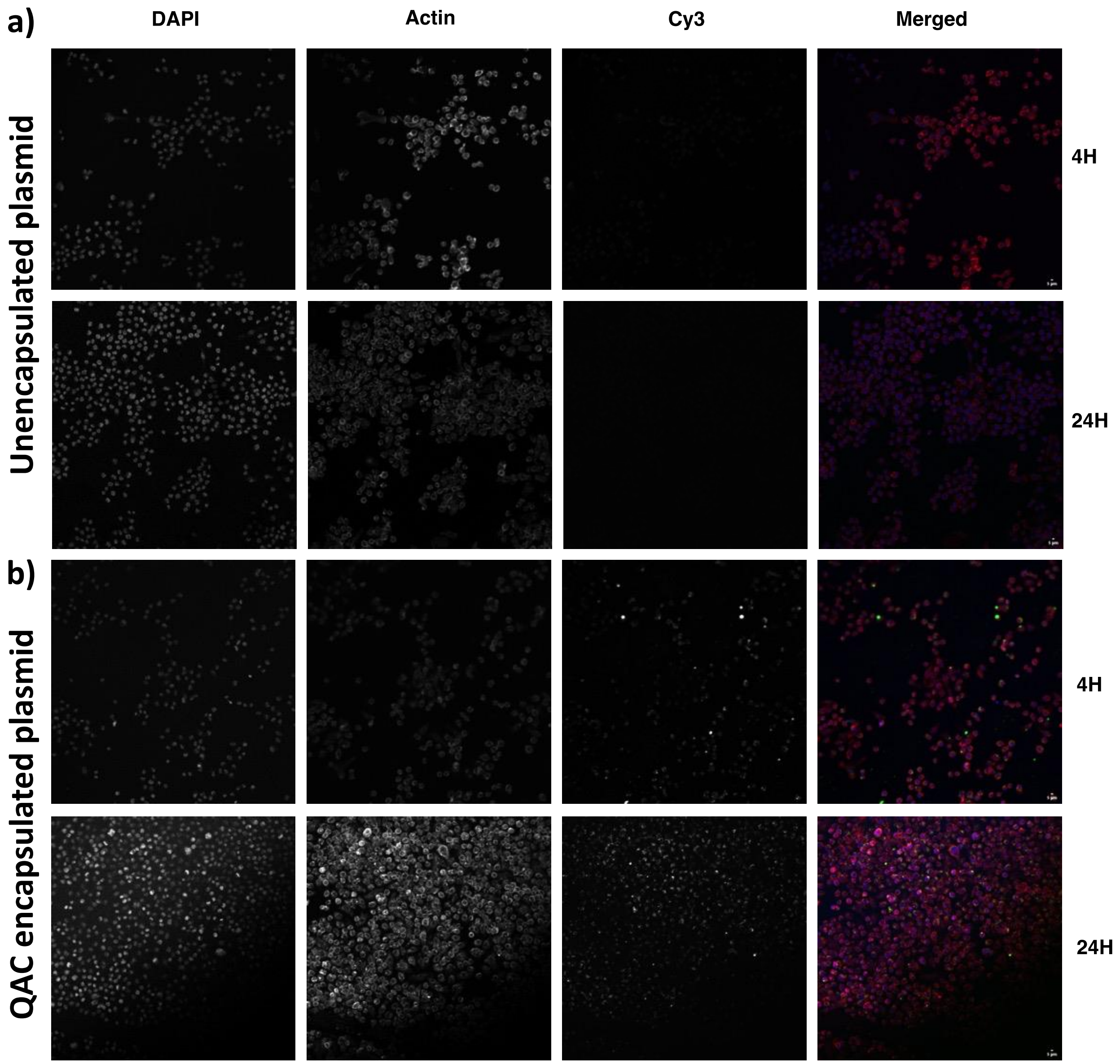

Supplementary Figure S5. Efficient internalization of QAC nanoparticles by J774 cells, 20X magnification. Cell monolayers were incubated with Cy3 labeled (A) unencapsulated or (B) QAC encapsulated labeled DNA (green) for 4 or 24 hrs and stained for actin (Alexa phalloidin 546, red). DAPI (blue) was used to stain the nucleus. Representative images were captured by LSCM. Scale bars = 5 μm.
